# A fine-scale multi-step approach to understand fish recruitment variability

**DOI:** 10.1038/s41598-020-73025-z

**Published:** 2020-09-30

**Authors:** Pablo Brosset, Andrew Douglas Smith, Stéphane Plourde, Martin Castonguay, Caroline Lehoux, Elisabeth Van Beveren

**Affiliations:** Fisheries and Oceans Canada, Maurice Lamontagne Institute, 850 Route de la Mer, Mont-Joli, QC G5H 3Z4 Canada

**Keywords:** Marine biology, Phenology

## Abstract

Recruitment is one of the dominant processes regulating fish population productivity. It is, however, notoriously difficult to predict, as it is the result of a complex multi-step process. Various fine-scale drivers might act on the pathway from adult population characteristics to spawning behaviour and egg production, and then to recruitment. Here, we provide a holistic analysis of the Northwest Atlantic mackerel recruitment process from 1982 to 2017 and exemplify why broad-scale recruitment–environment relationships could become unstable over time. Various demographic and environmental drivers had a synergetic effect on recruitment, but larval survival through a spatio-temporal match with prey was shown to be the key process. Recruitment was also mediated by maternal effects and a parent–offspring fitness trade-off due to the different feeding regimes of adults and larvae. A mismatch curtails the effects of high larval prey densities, so that despite the abundance of food in recent years, recruitment was relatively low and the pre-existing relationship with overall prey abundance broke down. Our results reaffirm major recruitment hypotheses and demonstrate the importance of fine-scale processes along the recruitment pathway, helping to improve recruitment predictions and potentially fisheries management.

## Introduction

Recruitment is a key component of the productivity of most marine fish species. Despite decades of research and some seminal hypotheses (e.g., critical period^1^; match–mismatch^2^), we still barely account for the observed variability in many stocks, and as such, it is often dubbed ‘the recruitment problem’^3^. This lack of understanding might, in part, be because the vast majority of studies have only focused on relationships between estimates of recruitment and spawning stock biomass (SSB) or broad-scale oceanographic indices due to data limitation^4,5^, thereby ignoring several important steps along the pathway leading to recruitment (e.g., spawning behaviour, egg production and/or early life stages survival^6,7^). Consequently, there are few examples exploring how the interplay between the biological traits of the adult population, the resulting spawning aspects (i.e., location, timing, and duration), and the environment experienced by adults and larvae determines recruitment to the spawning stock^4^.

Recruitment is the result of the number of eggs spawned and their survival. Total egg production (TEP) is known to be influenced by stock demographics, including SSB, age structure, and maternal body condition^8^. Specifically, older or fatter females usually produce disproportionally more eggs relative to younger smaller individuals. Following this, early life stage survival is a critical phase in the recruitment process^1^. Higher quality offspring, produced by larger and fatter females, might be more likely to survive (maternal effects^9,10^), as they can experience lower mortality rates when facing food shortage^11^ or predation^12^. However, the key element in larval survival is often the availability of food^13^, as prey need to be available in sufficient quantities at the right spatio-temporal scale (match–mismatch hypothesis^2^; ocean triads^14^; stable ocean hypothesis^15^). These three hypotheses relate fish recruitment strength to the temporal overlap between larval production and food availability (match–mismatch), the concomitance of food enrichment, concentration and retention processes (ocean triads), and to the concentration of fish larvae and plankton in a stratified water column (stable ocean hypothesis). Although these hypotheses are well-established, they have only rarely been verified due to a lack of concomitant larval and prey data at adequate temporal and spatial scales^16,17^.

The process described above can hardly be captured in a single relationship. Indeed, many of the key processes occurring during spawning and early life history are spatially and temporally constrained, and common broad-scale proxies such as climate indices might not always adequately represent the local conditions and processes such as to be meaningful. Many studies using such indicators have noted a breakdown or change over time of previously established relationships^18–22^. Although an apparent change in these relationships might, for instance, be caused by climate change, explanations for a discrepancy can only rarely be given as they do not shed light on the underlying mechanisms^18^. For instance, a potential driver (e.g., plankton abundance) might not seem significant anymore because its internal dynamics changed^23^. Furthermore, it is not always clear whether the breakdown of a relationship was caused by a poor initial quantification (e.g., through a spurious relationship, inaccurate variables or a previously neglected driver), a true ecosystem change or both.

The northern contingent (i.e., the “Canadian” stock) of Northwest Atlantic mackerel (*Scomber scombrus*, henceforth mackerel) is a good example of a stock for which the factors identified to drive recruitment have changed over the years. Mackerel recruitment has first been linked to zooplankton biomass (as a proxy of mackerel larvae prey production^24^), but the increasing contribution of *Calanus hyperboreus* (that does not produce eggs and nauplii available to mackerel larvae) to zooplankton biomass in the 2000s induced a breakdown of this relationship^23^. The former relationship was replaced by a refined correlation with copepod egg production (as a proxy of preferred mackerel larval prey abundance^23,25,26^), but Castonguay et al.^23^ predicted a strong 2006 cohort which never materialised. Later, recruitment was associated with factors such as zooplankton phenology and community structure, demonstrating the importance of a temporal match-mismatch^27,28^, but this study was based on composite environmental indices which had limited use for recruitment projections. Consequently, there is a need to understand the finer-scale processes involved in the transience of mackerel recruitment–environment relationships and to develop mackerel recruitment proxies relevant for management purposes.

The main purpose of this study was to provide a holistic view of the mackerel recruitment pathway by considering it as a multi-step process from spawners to recruits with particular attention on improving the spatio-temporal resolution of the modeled relationships. First, we analysed how spawning behaviour (i.e., timing, duration, and location) and egg production were influenced by demographic and environmental factors, and how these initial conditions, together with larval survival drivers, affected recruitment from 1982 to 2017. We therefore not only considered the importance of larval prey abundance, but also its availability, expressed as the spatial and temporal match with spawning sites, as well as causes of potential factors decoupling the relationships. Secondly, we contrasted our pathway-to-recruitment approach with previous studies which focussed only on the numbers of recruits. By doing so we aimed to show how our perspective on recruitment dynamics is shaped by the available data, and why some previously established recruitment–environment relationships might appear transient.

## Material and methods

**Figure 1:**
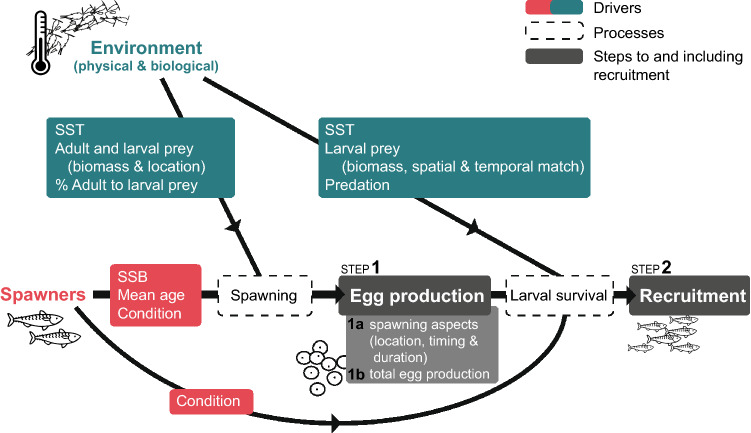
Conceptual framework of the pathway from spawners to recruits and the underlying mechanisms investigated (stock demographic structure and environmental conditions in red and green, respectively).

To investigate the pathway from adult population characteristics to spawning behaviour, egg production, and ultimately to recruitment (Fig. 1), we used three data sources; an egg survey (for estimates of egg distribution, total egg production, and environmental variables), biological samples of the commercial fishery (for estimates of spawning duration and peak, and maternal body condition), and stock assessment outputs (for estimates of age-1 recruits, spawning stock biomass and age structure).

### Egg survey data

#### Sampling

Mackerel enter the southern Gulf of St. Lawrence (sGSL, Eastern Canada) in early June each year to spawn, after overwintering along the north-eastern US continental shelf (from Sable Island to the Mid-Atlantic Bight^29,30^). Each year, Fisheries and Oceans Canada (DFO) conducts a 2-week long mackerel egg survey in the sGSL (a 65-station fixed grid 20 nautical miles apart spanning the dominant mackerel spawning area) around the average mackerel peak spawning date of June 21st. Over this period, a large fraction of spawning occurs and the survey is therefore believed to reflect appropriately spawning intensity and spatio-temporal properties. Stations consist of double oblique tows using 61-cm Bongo nets with 333 µm mesh size and flowmeters carried out on board a research vessel at a speed of 2.5 knots from 0 to 50 m depth to estimate daily and total egg production while also measuring physical and biological oceanographic variables (see further details in SI Appendix A). This survey has been carried out consistently since 1982, except for no surveys in 1995 and 1997. Several indices are derived from this mackerel egg survey: total egg production, egg distribution, water temperature, and zooplankton biomass, species composition, abundance, and distribution.

#### Total egg production and distribution

Annual total egg production was calculated according to a standard DFO protocol based on the Daily Egg Production Method^31^. Stage 1 (spawned less than 24 h ago) and 5 (i.e., damaged stage 1 eggs) egg counts were standardized by the volume of filtered water and the depth of the sampled water column to provide egg densities per station (number m^−2^). These numbers were then adjusted for incubation time^32^ to obtain daily egg production point estimates. Spatial interpolation was done across a grid of 3320 coordinates using ordinary kriging to calculate a mean daily egg production estimate per grid cell, which was extrapolated to the surface area sampled. Annual egg production estimates were obtained by dividing by the proportion of reproductively active fish at the median date of the survey. This latter value, along with peak spawning date and spawning duration was calculated using a logistic model describing the daily evolution of the gonadosomatic index, based on corresponding biological data (see further details in Doniol-Valcroze et al.^31^, and in “Commercial fishery sampling”).

To examine the potential inter-annual spatial mismatch between spawning location and the optimal habitat for larvae, we calculated the spatial extent (spawning area) and the position of the centre of gravity (spawning longitude and latitude) of spawning for each year in the time series. The spatial extent of egg production was determined using an *α*-convex hull on stations where eggs were present^33^. The centre of gravity of total egg production was calculated by taking the arithmetic mean of the coordinates of each station weighted by their individual observed egg production.

#### Environmental indices

Sea surface temperature (SST, °C) directly affects early life stage growth and survival^7^, but might also have an indirect effect on recruitment through adult spawning behaviour, as mackerel generally spawn between 8 and 15 °C^34^. Therefore, we produced an SST index by averaging June CTD-measured mean water temperatures in the first 10 m over stations, where the majority of mackerel eggs and larvae occur^35^.

We hypothesized that the main adult mackerel prey (i.e., *C. hyperboreus* and capelin, *Mallotus villosus*^36^) might be influential as well, as they may affect spawning location and therefore be an indirect driver of recruitment. Capelin is despite its importance as prey in terms of weight^36^ not considered as a potential driver of spawning location, because its consumption by mackerel is infrequent, only important to the larger mackerel and likely opportunistic. As such, habitat selection is most likely to be related to copepod abundance and we developed spatial, biomass, and composition indices in June in the sGSL only for *C. hyperboreus*. As a proxy of adult mackerel prey location, we computed the annual centre of gravity of *C. hyperboreus* biomass (latitude and longitude) with the same methodology used for total egg production. Also, we estimated the total *C. hyperboreus* biomass (mg m^−2^) in the sGSL^37^. The percentage of *C. hyperboreus* biomass relative to the total *Calanus* spp. biomass (% *C. hyp.*) was calculated as we hypothesized that changes in *C. hyperboreus* proportion may have influenced adult mackerel feeding behaviour and thus spawning locations.

Mackerel larvae mainly feed on the early life stages (eggs, nauplii, and young copepodites) of *C. finmarchicus*, *Pseudocalanus* spp. and *Temora longicornis*^25^*.* The copepod daily egg production (CEDP, µg egg carbon L^−1^ d^−1^) of these three copepod taxa, calculated based on adult female abundance and species-specific per capita daily egg production (see details in the SI Appendix A), was previously recognized as a good predictor of mackerel recruitment^23–25^. High larval prey abundance might, however, be irrelevant when there is a temporal or spatial mismatch with larval distribution. An annual (y) index of a temporal match was therefore calculated in June in the spawning area as the proportion of older stage 6 female *C. finmarchicus*, producing prey for mackerel early life stages, with respect to the number of younger immature copepodite stages 4 and 5^26^ (Eq. 1).

1 $${Temporal\ match}_{y}=100\%\times {N}_{C. fin female}/{N}_{C. fin stages 4-5}$$

Higher percentages of stage 6 female copepodites during mackerel spawning (i.e., a later development of the plankton community) should improve the temporal match between hatching and the availability of prey for emerging larvae^26^. This same index could not include *Pseudocalanus* spp. and *Temora longicornis* as only data for stage 6 adults were available. *C. finmarchicus* is, however, considered to be a good indicator of the overall zooplankton phenology in spring and early summer in the sGSL and should also reflect *Pseudocalanus* spp. and *Temora longicornis* phenology^27^. An annual index of a spatial match between mackerel egg distribution and their near-future prey was determined as the sum of mackerel daily egg production (DEP) at stations (s) with sufficient prey (i.e., copepod daily egg production above a threshold value) divided by the daily egg production of mackerel over all stations (Eq. 2).

2 $${Spatial\ match}_{y}=100\%\times {\sum }_{s=1}^{S>threshold}{DEP}_{s,y}/{\sum }_{s=1}^{S}{DEP}_{s,y}$$

The threshold copepod daily egg production value was determined as the 25th quantile of values measured for all years and stations, which excludes zero and near-zero prey availabilities unlikely to be able to support larval survival. This index of spatial match captures a combined effect of the abundance and distribution of the prey in relation to the distribution of the fish eggs. Note that due to the availability of taxonomic zooplankton data, *Pseudocalanus* spp., *Temora* spp., *C. finmarchicus* and *C. hyperboreus* data and hence all indices derived from it were available for only 21 years (but covering the entire span of the time series; 1982, 1985, 1987, 1990, 1993, 1996, 1999, 2000, 2003 and 2006 to 2017). Spatial and temporal match–mismatch proxies were based on a match with the mackerel eggs rather than the early larval phase. We expect this to introduce little noise as the development time of mackerel eggs is typically less than 6 days and mackerel larval development is fast (about 20 days^32^). All the environmental variables used and the associated hypotheses are summarized in Table 1.

**Table 1:** Summary of all the hypotheses tested along the pathway from spawners to recruits and associated references.

Hypothesis	Variables used	References
**Step 1a. Spawning aspects**		
Environmental effects		
SST determines available spawning area and peak of spawning	SST	^59^
Spawners prey mean location and distribution influences spawning mean location and area	*Calanus hyperboreus* mean location (lat/long) and area	^60^
*Calanus* spp. community composition influences area, duration and peak of spawning	The percentage of *C. hyperboreus* (weight) in the *Calanus* spp. group	^60^
Spawner prey biomass influences spawning duration	*Calanus hyperboreus* biomass	^8,60^
Spawning stock effects		
Spawning stock biomass affects spawning area, mean location, timing and duration	SSB	^59^
Maternal effects on egg spreading and spawning timing	Spawner mean age Spawner body condition	^10,38^
**Step 1b. Total egg production**		
Spawning stock effects		
Maternal effects on fecundity	Maternal body condition (*K*_*n*_)	^10,38^
**Step 2. Recruitment**		
Spawning aspects		
Spawning mean location, area and duration influence the probability to encounter larvae favorable conditions and recruitment	Spawning mean location (lat/long), area and duration	^8^
Total egg production determines recruitment strength	Total egg production	^57^
Environmental effects		
SST increases the probability of larval survival	SST	^61^
Predators affect larval survival	Spring herring biomass	^36^
Early life stage prey quantity affects larval survival	Copepods egg daily production (CEDP, *C. finmarchicus, Pseudocalanus* spp. and *Temora* spp.)	^23^
Early life stage prey availability in space and time affects larval survival (spatial and temporal match/mismatch)	% of mackerel eggs in stations with above threshold plankton quantity (spatial match proxy) % of stage 6 female *C. finmarchicus* with respect to younger immature copepodite stages (temporal match proxy)	^16,17^
Spawning stock effects		
Maternal effect on egg and larval quality	Maternal body condition (*K*_*n*_)	^8^

### Commercial fishery sampling

Adult mackerel samples are collected annually by DFO from the commercial fishery. The sampling covers the entire spawning area and period (thrice a week) and on average 4998 (range 421–14,858) individual fish are analysed each year. We used this data to calculate the annual peak spawning date (spawn. peak), spawning duration (spawn. duration), and maternal body condition.

Peak spawning date and duration were calculated each year based on the fit of a logistic model of the daily evolution of the gonadosomatic index. The mean value of the derived symmetrical probability density function was defined as the peak spawning day and the time between the 2.5% and 97.5% quantiles was estimated to represent the spawning duration in days.

As relatively fatter individuals might spawn more and higher quality eggs^38^, mature females (i.e., reproductive stages 3–8^39^) sampled between their arrival in the sGSL and June 21st (the average peak spawning date) were selected to investigate the potential influence of pre-spawning fat reserves on total egg production and recruitment with the relative body condition index (*K*_*n*_^40^, Eq. 3):

3 $${K}_{n}=\frac{W}{{W}_{r}}$$

where *W* is the observed somatic weight (g) of an individual and *W*_*r*_ the predicted weight of an individual of a given fork length (*FL*, cm) calculated with *W*_*r*_ = α*FL*^β^ (α and β are nonlinear least-squares regression parameters).

### Mackerel SSB, recruitment and age structure

Annual mackerel SSB, recruitment residuals and an index of age structure were derived from an age-structured state-space stock assessment model applied to the period 1968–2018^28^. Note that the model was calibrated using an SSB index directly calculated from total egg production. In the assessment model, a two-parameter Beverton-Holt stock-recruitment relationship was used to estimate annual recruitment (abundance at age 1), and the residuals of this relationship were used in subsequent analyses (R_res_). An indicator of the annual age structure was considered as bigger, older mackerel spawners (> age 5) are known to have a greater fecundity, and spawn in different spatial and temporal niches than younger females^35,41^. Mean biomass-weighted age (*MA*) was calculated using mature biomass-at-age (${SSB}_{a}$) as follow in the Eq. (4):

4 $$MA=\frac{\sum_{a=1}^{a=10}(a{SSB}_{a})}{\sum_{a=1}^{a=10}{SSB}_{a}}$$

*MA* was based on biomass rather than abundance to better reflect the stock’s reproductive potential^42^.

Mackerel early life stages are prey for pelagic fish sharing the surface waters of the sGSL. Herring are, relative to other potential predators, dominant, widely distributed and known predators of mackerel eggs and larvae^36^. Hence, we used cumulated spring and fall herring model-derived annual biomass^43^ as a proxy of predation pressure on mackerel early life stages.

### Statistical analyses

#### Recruitment variability driven by spawning aspects and environmental gradients

We analysed the relationships between the successive steps leading to recruitment (spawning aspects, egg production and recruitment) and both demographic and environmental effects using generalised linear models (GLMs). All model configurations (response and explanatory variables) are given in Supplementary Table S2. Explanatory variables were normalized (i.e., by subtracting the mean and dividing by the standard deviation for each variable) to facilitate comparison of their respective effects (i.e., through their coefficients). When the response variable was R_res_ (with a 1-year lag), residuals were assumed to follow a Gaussian distribution with an identity link function, whereas for the other response variables a Gamma distribution with a log link function was used (as they can only take positive values^44^). Before performing GLM computations, collinearity between explanatory variables was measured using variance inflation factors (VIFs), considering a VIF threshold of 3^44^. Specifically, mackerel SSB and *MA* were highly correlated (Pearson correlation coefficient > 0.7, see Supplementary Fig S1), so distinct sets of GLMs testing SSB or *MA* on spawning aspects were used. A backwards model selection procedure was performed, choosing the model with the lowest Akaike’s information criterion corrected for small samples sizes (AICc). If independent models including either SSB or *MA* showed an AICc difference less than 2, both were reported. Assumptions of homoscedasticity and normality were checked using residual plots while assumptions of independence (to ensure no autocorrelation was present) were checked using correlograms. By replacing GLMs with generalized additive models, the same conclusions were reached and there were no indications of strong non-linear effects.

Variability in total egg production (TEP) could not be linked directly to SSB and *MA* using regression techniques, because of model circularity (a TEP derived SSB index was used to estimate SSB) and collinearity (SSB and *MA* are significantly correlated and difficult to disentangle). Although the relative effect size of both variables could not be measured, the positive link between them is well established in the literature (i.e., that larger, older fish produce more eggs^41^). We, therefore, focussed our efforts on the possible link between TEP per unit of biomass, thereby removing the effect of fish number- and weight-at-age, and maternal body condition. Furthermore, by working with stock–recruitment residuals, we removed in large part the intrinsically related process of TEP. That is, the stock–recruitment relationship is presumably created by the biological dependence of TEP on SSB, and subsequently of recruitment on TEP. This link was hence not explicitly considered, although being present. A Jackknife procedure was conducted to assess the consistency and robustness of the optimal models explaining recruitment residuals (see SI appendix A). Also, recruitment estimates are inherently dependent on the modelling choices^45^, and we verified that recruitment residuals obtained under different assumptions (i.e., through a Virtual Population Analysis, VPA^46^) were not differently explained by the considered variables (see SI appendix A for more details).

#### Stability of the recruitment-larval prey availability relationship

Since Castonguay et al.^23^, a different stock assessment model has been employed, resulting in new recruitment timeseries^47^. As a baseline for comparison, we, therefore, refitted the recruitment–CEDP relationship from Castonguay et al.^23^ with the updated estimates and including all years (1982–2017, linear modelling). We hypothesized that, with the addition of new years of data, potential changes in the performance of this quantitative food index (i.e., CEDP) in predicting recruitment would be driven by a temporal change in the relationship because of altering underlying mechanisms. The latter could manifest itself as changes in the spatial or temporal match between the CEDP and the spawning distribution (a proxy of larval distribution), i.e., the ‘effective’ prey availability. Thus, we examined whether changing larval prey availability in space and time, coupled with a changing mackerel larval quality (using adult *K*_*n*_ as a proxy), can explain residuals and the potential breakdown of the R_res_-CEDP relationship. Then, the drivers behind the spatial match-mismatch between mackerel eggs and larval prey were investigated. We considered maternal body condition, SST, and *C. hyperboreus* longitude (i.e., spawner prey). We also retained the relative abundance of *C. hyperboreus* in the *Calanus* spp. community (% *C. hyp.*), as this species does not produce eggs and nauplii available to mackerel larvae in the summer in the sGSL^37,48^ and appears to reduce abundance of *C. finmarchicus* early life stages (i.e., mackerel larval prey) through predation^49^. Thus, years with a large proportion of *C. hyperboreus* in the plankton community may display a larger mismatch between mackerel eggs and CEDP. A beta regression model was used to study the spatial match (as it is a proportion). All statistical analyses were conducted with R (version 3.3.2^50^).

### Ethical approval

This study was approved by DFO Research Ethics Board and conducted with methods in accordance with the Canadian Council on Animal Care (ISBN: 0-919087-43-4).

## Results

### Spawning aspects

**Figure 2:**
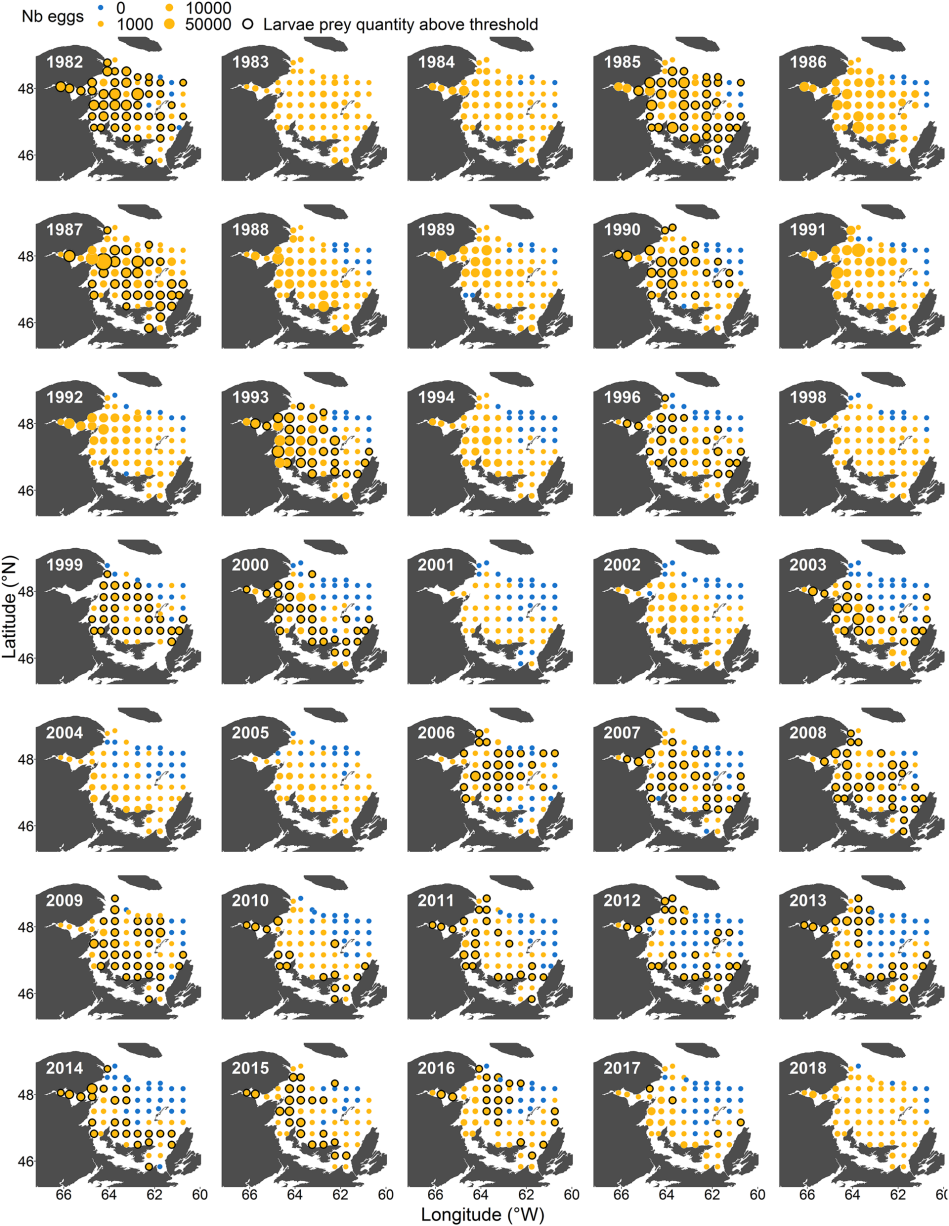
Maps of the annual egg production at each station between 1982 and 2018 in the southern Gulf of St. Lawrence. Blue dots indicate an absence of mackerel eggs while the size of the orange dots is proportional to egg production. For years with zooplankton data (1982, 1985, 1987, 1990, 1993, 1996, 1999, 2000, 2003, 2006–2017), black outlines indicate stations with larval prey quantity above the 25% overall quantile. There was no survey in 1995 and 1997.

**Figure 3:**
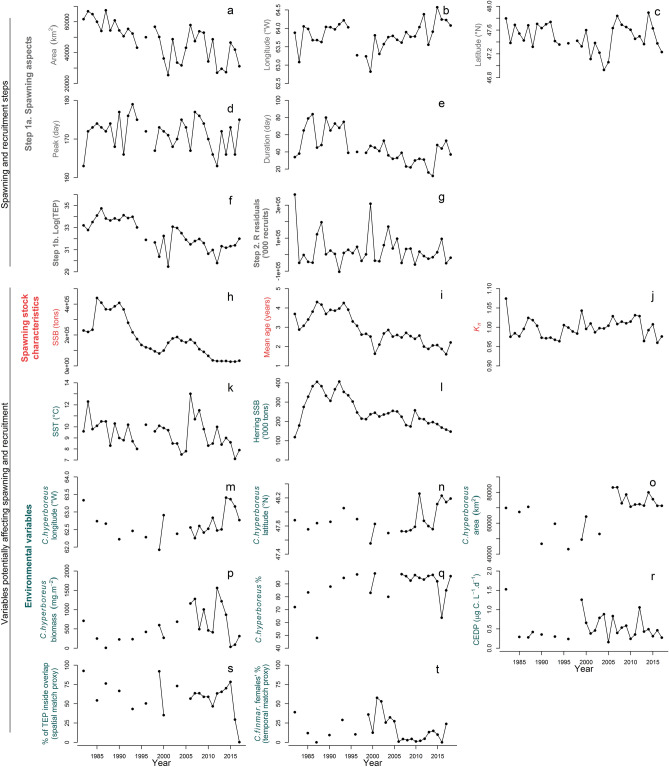
Time series (1982–2017) of the steps leading to recruitment (grey), the mackerel spawning stock characteristics (red) and the environmental conditions (green). TEP: total egg production; K_n_: female body condition; CEDP: copepod egg daily production.

**Figure 4:**
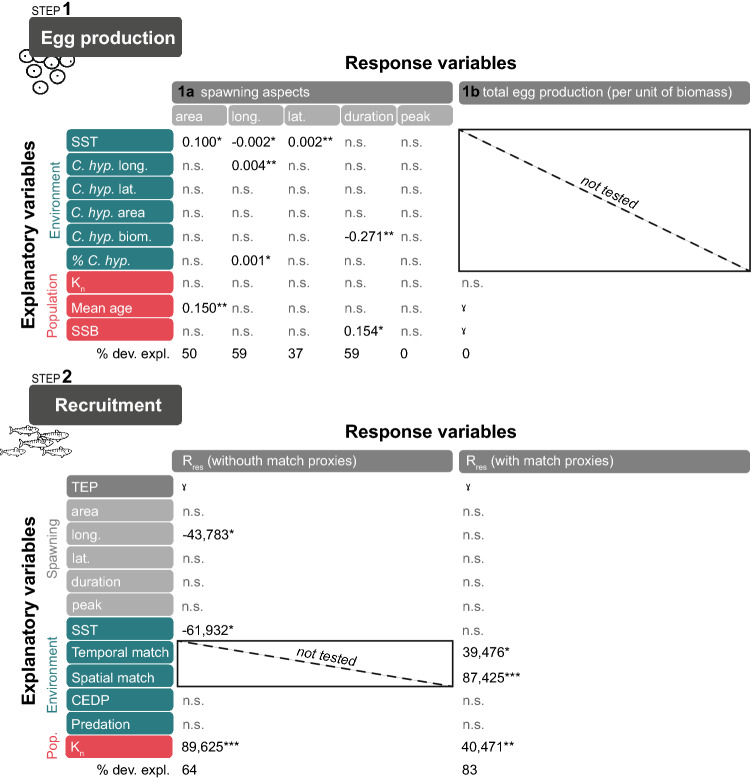
Coefficients of the significant drivers explaining mackerel spawning aspects (step 1a), total egg production (TEP, step 1b), and recruitment residuals (R_res_, step 2) in the southern Gulf of St. Lawrence between 1982 and 2017, retained in the optimal Generalized Linear Models (note that only 21 years are used for the spawning longitude/latitude models and when considering match proxies). ˠPositive effects that cannot directly be quantified (as TEP is strongly linked to SSB, see text). Significance levels are indicated with asterisks (***for p < 0.001, **for p < 0.01, and *for p < 0.05). *Calanus hyperboreus*: *C. hyp.*; the percentage of *C. hyperboreus* biomass relative to the total *Calanus* spp. biomass: % *C. hyp.;* female body condition: *K*_*n*_; total egg production: TEP; copepod egg daily production: CEDP. The units of the coeffients are equal to the unit of the response variable; area (km^2^), longitude (°W), latitude (°N), duration and peak (day), TEP (number m^−2^), and R_res_ (‘000 s recruits).

The spawning area at the beginning of the time series (1982–1989) extended across most of the study region (Fig. 2). However, after 1989, the spatial extent of spawning contracted, and after 2000, spawning had all but disappeared from the northeast area. As the spatial extent of spawning decreased, its interannual variability also became more pronounced (Fig. 3a). This contraction was significantly related to a decline in SSB and/or mean age (both are correlated, Fig. 3h,i) and lower SST (Fig. 3k) which explained 50% of the deviance (Fig. 4 step 1a, see also Supplementary Table S2 for details of models tested and Supplementary Fig S2 for the residuals’ correlograms). The mean location of mackerel spawning (i.e., spawning longitude and latitude) in the sGSL shifted eastward between 1995 and 1998 and progressed slightly westward again after 2000 (Fig. 3b). Until 2005, spawning also moved generally southwards, followed by a large shift north over the next 3 years to subsequently move southward again (Fig. 3c). The longitudinal position of the egg distribution was strongly associated with the center of biomass of *C. hyperboreus* (Fig. 3m), but also with SST (Fig. 3k) and the proportion of *C. hyperboreus* in the *Calanus* spp. community (Fig. 3q, 59% of the deviance explained, Fig. 4, step 1a). Latitudinal changes were, however, only significantly related to SST (Fig. 3k, 37% of deviance explained, Fig. 4, step 1a) and not to *C. hyperboreus* latitude (Fig. 3n) and area (Fig. 3o).

In contrast to the spatial aspects of spawning, the peak spawning date was relatively constant over time (Fig. 3d), and could not be significantly explained by any of the variables considered in this study (Fig. 4, step 1a). The duration of the spawning season, on the other hand, shrank between the 1990s and the beginning of the 2010s, before increasing slightly to values closer to the time series’ average after 2013 (Fig. 3e). Longer spawning periods occurred when the population’s biomass was larger (Fig. 3h) and its age structure less truncated (Fig. 3i), but *C. hyperboreus* biomass was lower (Fig. 3p, 59% of deviance explained, Fig. 4, step 1a).

### Total egg production

Egg production per station during the 1980s through to the early 1990s was often estimated at over 10,000 eggs, representing a total egg production (TEP) beyond 1.10^15^ eggs in the sGSL (Figs. 2, 3f). These numbers have however substantially decreased since, with a TEP in 2017 representing 10% of the historically observed maximum of 1986 (Fig. 3f). The TEP per unit of biomass was not significantly correlated with maternal body condition (*K*_*n*_, Fig. 4, step 1b).

### Recruitment

**Figure 5:**
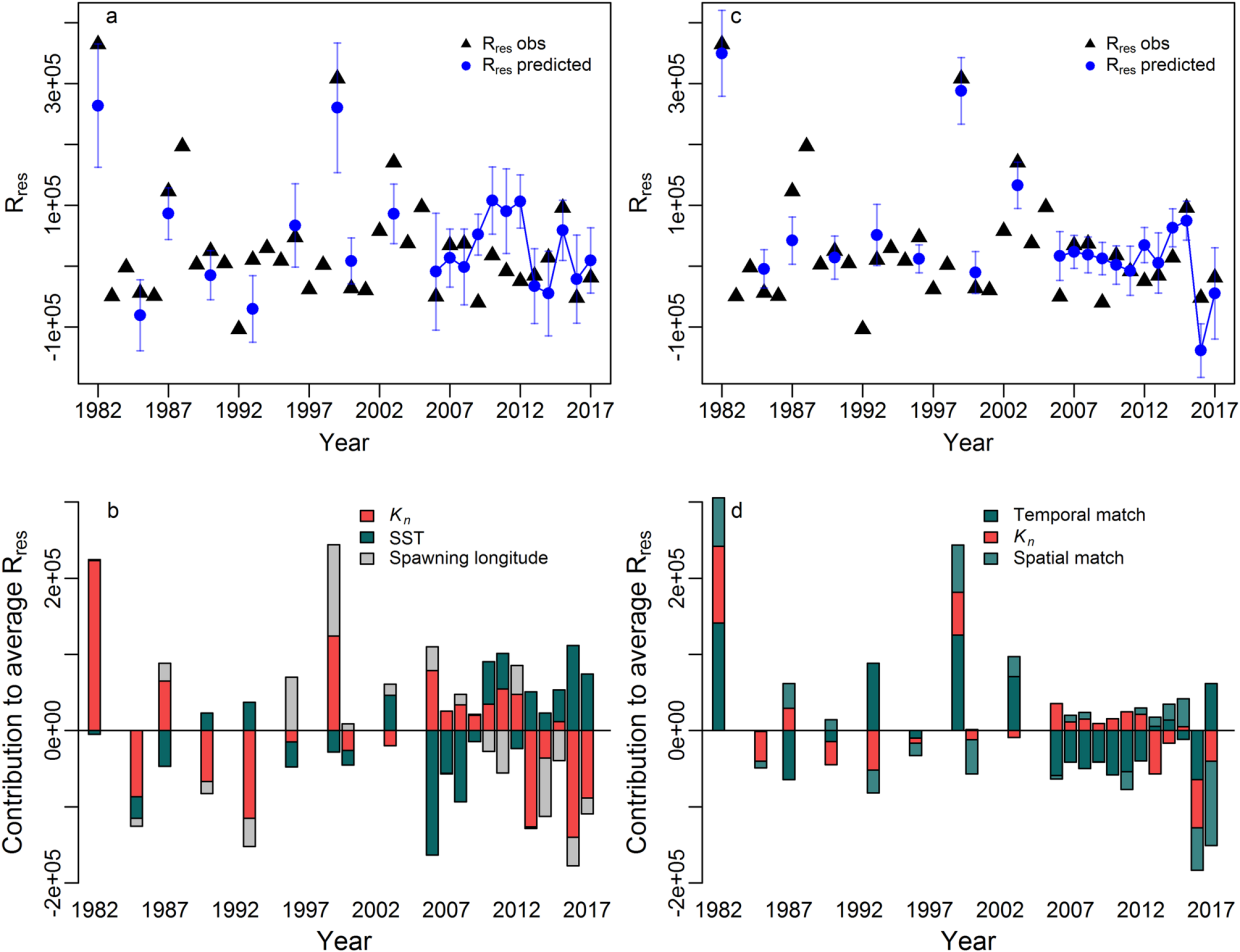
The output of the GLM models predicting one year lagged recruitment residuals with predicted versus observed plots (upper panels) and plots showing the contribution of the significant variables to the prediction of R_res_ (lower panels). GLM were fitted without (left) and with (right) match–mismatch proxies. The blue error bars indicate the 95% confidence interval. Drivers of recruitment are indicated by color (red = demographic, green = environmental, grey = spawning aspects).

The age-1 stock-recruitment residuals (R_res_) displayed large interannual variability with peaks in 1982, 1988, 1999, 2003 and 2015 (Fig. 3g). Without accounting for match–mismatch proxies, R_res_ was positively correlated with *K*_*n*_ (Fig. 3j) and negatively with SST and spawning longitude (Fig. 3b,k, 64% of variance explained, Fig. 4, step 2). The peaks of 1982 and 1999 were well predicted (Fig. 5a) and resulted from the eastern spawning longitude (related to adult prey location, see the previous section) and favorable fish *K*_*n*_ (Fig. 5b).

When the spatial and temporal match-mismatch proxies between mackerel eggs and larval prey were considered (Fig. 3s,t), they were both retained by the model in addition to *K*_*n*_ (83% of deviance explained, Fig. 4, step 2), whereas SST and spawning longitude were rejected. The 1982 and 1999 recruitment highs were still well predicted (Fig. 5c), but here by a strong temporal and spatial match of larvae with their potential prey, as well as by a good maternal body condition (Fig. 5d). All other variables, including the CEDP (i.e. larval prey abundance, Fig. 3r), herring SSB (Fig. 3l) or other spawning aspects (area, location and duration) did not explain R_res_ significantly. Jackknife analyses showed that the optimal models considering spatial and temporal match indices remained ranked top in almost all cases (Supplementary Fig. S3). In addition, GLMs using recruitment residuals from the VPA (Supplementary Fig. S4) also highlighted the spatial and temporal matches as significant to explain recruitment variability, but the deviance explained was weaker than those obtained with recruitment deviates from the current stock assessment model (62%).

### Stability of the recruitment-larval prey abundance relationship

**Figure 6:**
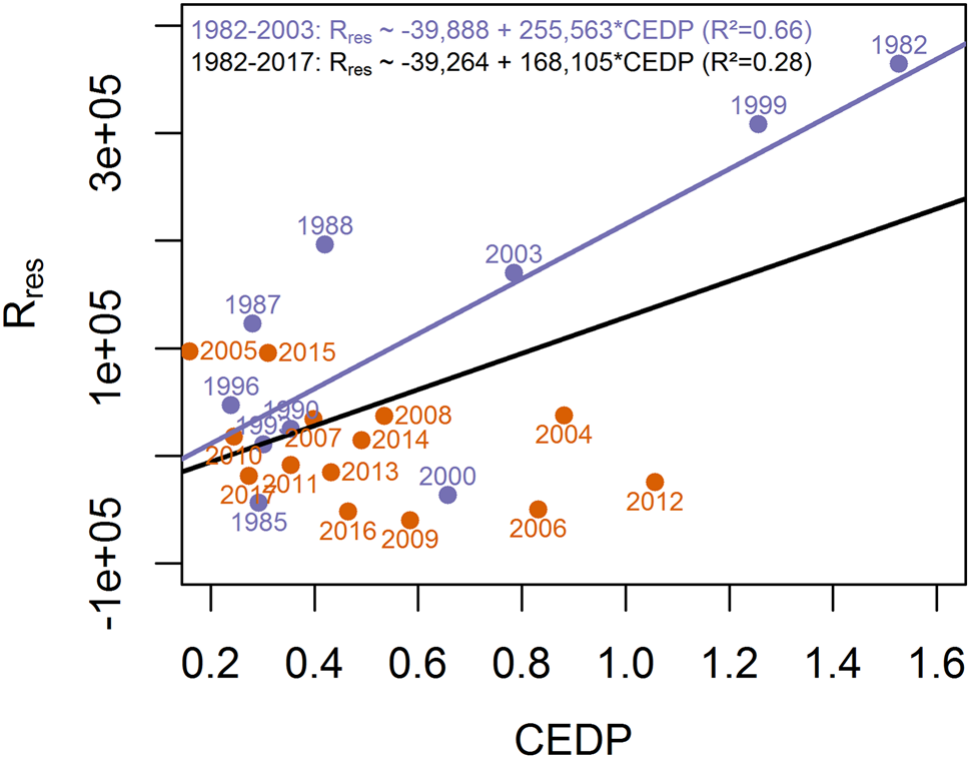
Significant linear relationships between residuals of mackerel recruitment (R_res_) and copepod egg daily production (CEDP). No significant relationship was detected for the 2004–2017 period.

The univariate R_res_-CEDP relationship previously established for mackerel from 1982 to 2003 was still significant when refitted from 1982 to 2017 (*p* < 0.05, Fig. 6). Yet, the CEDP index explained less than half the variance compared to the 1982–2003 period (*R*^2^ = 0.28 vs 0.70, Fig. 6). Indeed, a linear model focusing only on data from 2004 to 2017 was not significant (*p* > 0.05) and even suggested reversal in the direction of the relationship. The residuals of the full period relationship were explained by the spatial and temporal match of mackerel eggs with prey availability (R^2^ = 0.39). That is, the large negative residuals of the last decade, indicating lower recruitment than predicted solely by the CEDP, came from a poor spatial (*p* < 0.05, slope = 35,758) and temporal (*p* < 0.05, slope = 48,225) match. The spatial mismatch was in part produced by a poor maternal body condition (beta regression, *p* < 0.05, slope = − 0.58) and a higher percentage of *C. hyperboreus* relative to total *Calanus* spp. biomass (beta regression, *p* < 0.05, slope = 0.43; R^2^ = 0.38).

## Discussion

Our understanding of recruitment can only be as thorough as the scale and the number of the underlying processes and drivers studied. Through a holistic approach considering the entire pathway from spawners to recruits and the refinement of variables considered, our study provides new key insights on fish recruitment dynamics and demonstrated the multidimensionality of the process leading up to recruitment. Although mackerel stock biomass determines long-term average recruitment patterns, recruitment peaks were produced by high larval survival resulting from a positive spatio-temporal match of mackerel larvae with their prey, as well as optimal maternal body condition. Hence previously used estimates of prey abundance became a relatively poor proxy for food availability, as spatio-temporal mismatch caused the relationship to weaken with the addition of new years.

### What are the drivers of recruitment variability?

Our findings follow the common view that the level of extreme early life stage mortality will largely determine the recruitment outcome^5^. Food abundance is generally postulated to be the critical determinant in recruitment, as was so far already assumed for mackerel^23,24^. There is, however, a clear interaction between food abundance and its temporal and spatial availability that is often ignored, but which can be essential in understanding and predicting recruitment. Despite the importance of the match-mismatch hypothesis^2^, it has to our knowledge only been rarely demonstrated, whether on a spatial or temporal scale^17,51,52^ or both^62^. This should be noted in relation to the vast amounts of studies focusing on marine species recruitment variability^4^. We confirmed, for northwest Atlantic mackerel, that the best descriptors of larval food availability are proxies of the spatial and temporal match of larvae and their prey, rather than prey abundance, even if larger overall food abundances have been demonstrated to potentially compensate for small mismatches^13^.

We found that the temporal component of prey availability (i.e., the proportion of females in the *C. finmarchicus* population) had the strongest influence on recruitment estimates, a driver already identified using a more general zooplankton phenology proxy^27^. In particular, a temporal mismatch was related to the lower recruitment estimates during the last decade. During this recent period, the peak in prey availability occurred earlier in the season through advanced copepod reproduction and development, likely triggered by an earlier *C. finmarchicus* adult arousal from diapause^37^. At the time of mackerel hatching, copepodites would already have reached their late stages and would be too large to be consumed by young mackerel larvae. In principle, a temporal mismatch with prey could be buffered by a parallel shift in the timing of adult mackerel spawning. However, the timing of spawning was relatively constant and seemed independent of any of the factors investigated in this study (e.g., SST in the spawning area). Mackerel spawning timing may for instance rather be affected by the timing of arrival in the sGSL and thus by the winter-spring environmental conditions encountered in the overwintering and migration areas outside the Gulf of St. Lawrence. This may prevent spawner phenology to track the changing plankton phenology in the spawning area and would be an example of how global climate change disparately affects marine communities^53^.

Likewise, an optimal spatial match between mackerel larvae and their prey favored recruitment. This was important for the 2015 recruitment peak, estimated to be produced by a good spatial match while the temporal match and maternal body condition were around their long-term averaged value. Interestingly, the trade-off between maximising parental fitness and increasing future offspring survival is likely a crucial factor in determining a spatial match. Spawning habitat location was determined by thermal preferences and, in particular, by the location of adult mackerel prey rather than larval prey. Additionally, the spatial match between mackerel larvae and their prey significantly decreased when the copepod community was dominated by *C. hyperboreus* (the preferred adult food) and females had an overall poorer body condition. Thus, the recent higher proportion of *C. hyperboreus* in the *Calanus* community could have caused a decoupling between the larvae and their prey. Indeed, larger proportions of *C. hyperboreus* might reduce the local abundance of *C. finmarchicus* early life stages^49^ (i.e., mackerel larval prey), so that when adults track prey abundant areas, they move to areas that are increasingly suboptimal for larval development. Poor *K*_*n*_ might also lead to a stronger favoring of areas with high adult prey densities, which are not necessarily beneficial for larval development.

Recruitment peaks never occurred when females had a below-average body condition. As mentioned above, a poor body condition may make areas dominated by *C. hyperboreus* but with potentially lower larval prey abundance more attractive for adult mackerel, and hence disfavor recruitment. This shows that the effect of maternal body condition might not only act through increased offspring number and survival^10,38^ but perhaps also through behavioural change, supporting a larger importance of ‘parental condition’ in determining recruitment than traditionally considered^8,54^. The effects of other maternal effects on recruitment, such as age, are however harder to establish. For instance, age structure and biomass are often highly correlated (e.g., through overfishing) and their effects are hardly distinguishable. Nonetheless, changes in stock demography (and thus in ‘spawning strength’) could influence mackerel recruitment variability. Note for example that the spatial and temporal extent of spawning paralleled the truncation of the stock’s age structure and biomass (as for Japanese sardine^55^). Indeed, a temporal difference in the timing of spawning between age classes is known for mackerel, and fewer fish are likely to spawn over a reduced temporal and geographical range^41^. Because of the importance of the match-mismatch mechanisms shown here, age truncation is likely to diminish the probability of having a good match. This supports the role of fish population demographics as a buffer to recruitment, as already demonstrated for several fish stocks^56^. Further work, however, will be needed to better understand and quantify the effect of age structure versus density-dependant processes on spawning behaviour^57^. Note that the absence of an effect of female body condition on egg numbers was unexpected and might be a statistical artefact or result in part from the consideration of total egg production per unit of biomass.

### What causes our perception of the recruitment–environment relationship to change?

The majority of recruitment–environment relationships breakdown when they are updated with new data^19^, which can have serious consequences for fisheries management^4^. For mackerel, the explanatory power of the relationship between recruitment and a previously used larval prey proxy (CEDP) weakened critically with environmental changes. Specifically, the mackerel recruitment–CEDP relationship is now twice weaker than previously in Castonguay et al.^23^ and does not fit the more recent data (2004–2017). Between 1982 and 2003, a high CEDP might have implicitly reflected a good spatio-temporal match between mackerel larvae and their prey. In recent years, however, food was abundant but became less available because of an earlier *C. finmarchicus* development and stronger dominance of *C. hyperboreus*, to which mackerel did or could not sufficiently adapt, resulting in an increased spatio-temporal mismatch. The 2006 mackerel peak forecasted by Castonguay et al.^23^ -based solely on the CEDP- consequently failed to materialize, as the spatial and especially the temporal matches were weak, and prey availability was thus probably insufficient to promote significant larval growth and survival. This case study illustrates the advantage of considering the synergetic effects of all the above fine-scale mechanisms. It also confirms that the use of more general variables, such as overall prey abundance and large-scale climate indices, might be too noisy to reliably predict fish recruitment, explaining their tendency to appear volatile over time^18–20^.

### Implications for mackerel management

Our perception of recruitment variability might be continuously evolving, but a more detailed understanding helps expose the strengths and weaknesses of the presumed relationships. The strong and intricate relationships highlighted here should help improve recruitment forecasting. For instance, key fine-scale zooplankton variables are collected during the spawning period and can be used for one year ahead predictions as soon as the samples are processed in the laboratory. Furthermore, because mackerel spawning peak seems fixed and hence appears relatively inefficient in adjusting to changes in the environment and also considering the low spatio-temporal buffering at the current depleted stock level, it is unlikely that regular peaks in recruitment will occur in the future if the current environmental conditions persist. Such information can assist the otherwise largely subjective choice used for a recruitment projection method, which has a large impact on the scientific advice to fisheries management^58^. We advocate for the collection of more fine-scale data and whenever possible to conduct multi-step analyses of the recruitment process, in the hope of reducing ‘the recruitment problem’. Consequently, fine-scale mechanisms should be of central interest in future recruitment-related studies and for the inclusion in a stock assessment framework, ultimately optimizing conservation and management.

## Supplementary information

Supplementary file1

## Data Availability

The datasets analysed during the current study are available from the corresponding author on reasonable request.
