# Supplementary material for: A fine-scale multi-step approach to understand fish recruitment variability
Source: Sci Rep. 2020 Sep 30;10:16064. doi: 10.1038/s41598-020-73025-z (PMC7527453; doi:10.1038/s41598-020-73025-z)
Supplement: Supplementary file 1 — Supplementary file1 [file 41598_2020_73025_MOESM1_ESM.docx]

**Supplementary Information**

**A fine-scale multi-step approach to understand fish recruitment variability**

Pablo **BROSSET^1^,** Andrew Douglas **SMITH^1^,** Stéphane **PLOURDE^1^,** Martin **CASTONGUAY^1^,** Caroline **LEHOUX^1^,** Elisabeth **VAN BEVEREN^1^**

*^1^ Fisheries and Oceans Canada, Maurice Lamontagne Institute, Mont-Joli, Québec G5H 3Z4, Canada*

**Appendix A.**

**Methods:**

**Egg survey sampling scheme**

At each station, plankton samples were collected from the upper water column (surface to 50 m or down to 5 m off the bottom for shallower stations) using two 61 cm diameter bongo nets (333 µm mesh size) deployed at a 45° angle. Each oblique tow lasted a minimum of 10 minutes, undulating from surface to maximum depth at a speed of 2.5 knots or 1.3 m.s^-1^. The filtered volume of water, temperature, and salinity were measured using a modified CTD that was also equipped with an accelerometer to monitor the pitch and angle of the bongo nets. Once onboard, the nets were washed with salt water to concentrate the plankton samples in the cod ends of the nets before preserving samples from one net in 4% formaldehyde and those from the second net in 100% concentrated ethanol to serve as backups^1^. In the laboratory, mackerel eggs, larvae and plankton were sorted and counted by way of a ‘Dutch shuffle’^2^. All organisms were identified to the lowest possible taxonomic level (based on^3–5^) and mackerel eggs were sorted and counted by stage of development^6^.

**Daily egg production of *Calanus finmarchicus*, *Pseudocalanus* spp. and *Temora longicornis***

To assure continuity in data collection between 1982 and 2017, the stations corresponding to the western part of the “Iles de la Madeleine” sampling line were retained (stations 5.3, 5.4, 6.4, 7.3, 7.4, 8.3, 8.4 from 1982 to 1999 in the mackerel egg survey and stations TIDM 6 to 10 from 2001 to 2017 in the Atlantic Zone Monitoring Program^7^). The estimated daily egg production of major copepod species (CEDP, µg.C.L^-1^.d^-1^) at each station was calculated as follows:

${CEDP}_{y,s}= \frac{\sum_{t=1}^{T} N_{fem,y,s}^{t}*{DEP}^{t}*W_{E}^{t}}{1000}$,

where $N_{fem,y,s}^{t}$ is the female abundance of copepod taxon *t* (see Table A1), ${DEP}^{t}$ is the taxon specific daily egg production rate, $W_{E}^{t}$ is the taxon specific egg carbon weight, and 1000 is a volumetric correction factor (1 cubic meter). The daily egg production per year corresponds to the sum of the estimated daily egg productions for all the stations considered (${CEDP}_{y}= \sum_{s=1}^{S} {CEDP}_{y,s}$).

Table S1. Copepod species included in the calculation of the CEDP (copepod egg daily production) and their variables. DEP = copepod daily egg production rate ; W_E_ = egg carbon weight.

| Copepod taxon (*t*) | $DEP$ (eggs.female^-1^.d^-1^) | $W_{E}$ (µg.C.egg^-1^) |
| --- | --- | --- |
| *C. finmarchicus* | 50^8^ | 0.23^9^ |
| *Pseudocalanus* spp. | 5^10^ | 0.14^11^ |
| *T. longicornis* | 30^12^ | 0.04^11^ |

**Model validation (Jackknife procedure and alternative recruitment estimates)**

For the Jackknife procedure, each data point in the time series was successively omitted and all possible GLMs were refitted to the remaining data set. These models were then sorted according to AICc to determine whether the optimal model remained the top‐ranking model. The variance explained was also calculated for comparison.

The estimated SSB and recruitment from a previously fitted (1982-2014) virtual population analysis (VPA) were obtained to verify the sensitivity of results to stock assessment models assumptions (i.e., to investigate if the same explanatory variables are identified to explain the variability of recruitment estimates coming from different models). As the VPA did not assume any stock-recruitment relationship^13^, we used mackerel recruitment residuals (1982-2013) as other recruitment proxies. GLM using this alternative recruitment time-series as response variable was achieved with the same explanatory variables that the ones used to explain the Beverton-Holt recruitment residuals coming from the censored catch models.

**Appendix B.**

Table S2. Details of the GLM models between 1982 and 2017. Separate models were fit when including stock mean age or spawning stock biomass (both are correlated), or using time series of different length (either without plankton based variables, i.e., all years) or with plankton based variables, i.e., only 21 years). Only optimal models for a given response variable and time series length and alternative models with AICc less than 2 higher than the best model are presented. Optimal models (with the lowest AICc) are in bold. *p*-value are indicated by: * < 0.05; ** < 0.01; ***<0.001. AICc = Akaike’s Information Criterion corrected for small samples sizes, DEV = percentage of deviance explained, *K*_n_ = mackerel body condition, CEDP = copepods egg daily production.

| **Years considered** | **Model** | **AICc** | **DEV** |
| --- | --- | --- | --- |
|  | ***Step 1a. Spawning aspects*** |  |  |
| 1982-2017 | *Spawn. longitude* ~ SSB | 34.63 | 1 |
|  | *Spawn. longitude* ~ Mean age | 34.69 | 1 |
| 1982-2017 (only 21y) | ***Spawn. longitude*** **~ SST* + % *C. hyp*.* + *C. hyp*. longitude**** | **7.33** | **59.67** |
| 1982-2017 | *Spawn. latitude* ~ SSB | -20.07 | 16 |
|  | *Spawn. latitude* ~ Mean age | -19.40 | 14 |
| 1982-2017 (only 21y) | ***Spawn. latitude*** **~ SST **** | **-23.57** | **37** |
| 1982-2017 | *Spawn. area* ~ SSB** | 729.84 | 32 |
|  | *Spawn. area* ~ Mean age** | 729.26 | 33 |
| 1982-2017 (only 21y) | *Spawn. area* ~ SSB** + SST* | 447.89 | 48 |
|  | ***Spawn. area* ~ Mean age** + SST*** | **447.12** | **50** |
| 1982-2017 | *Spawn. duration* ~ SSB* | 279.22 | 41 |
|  | *Spawn. duration* ~ Mean Age | 286.33 | 28 |
| 1982-2017 (only 21y) | ***Spawn. duration* ~** **SSB* + *C. hyp.* biomass**** | **168.37** | **59** |
|  | ***Step 1b.TEP per unit of biomass*** |  |  |
| 1982-2017 | *TEP* ~ *K*_n_ | 2375.40 | 8 |
|  | ***Step 2. Recruitment*** |  |  |
| 1982-2017 | *R_res_* ~ Mean age | 933.37 | 4 |
| 1982-2017 (only 21y) | ***R_res_* ~ *K_n_*** + spatial match* + temporal match***** | **517.88** | **83** |
|  | ***Additional models*** |  |  |
| 1982-2017 (only 21y) | ***Spatial match* ~ % *C. hyp.*** + *K_n_**** | **-4.25** | **38** |
| 1982-2017 (only 21y) | ***R_res_ -CEDP residuals* ~ spatial match* + temporal match*** | **534.46** | **39** |


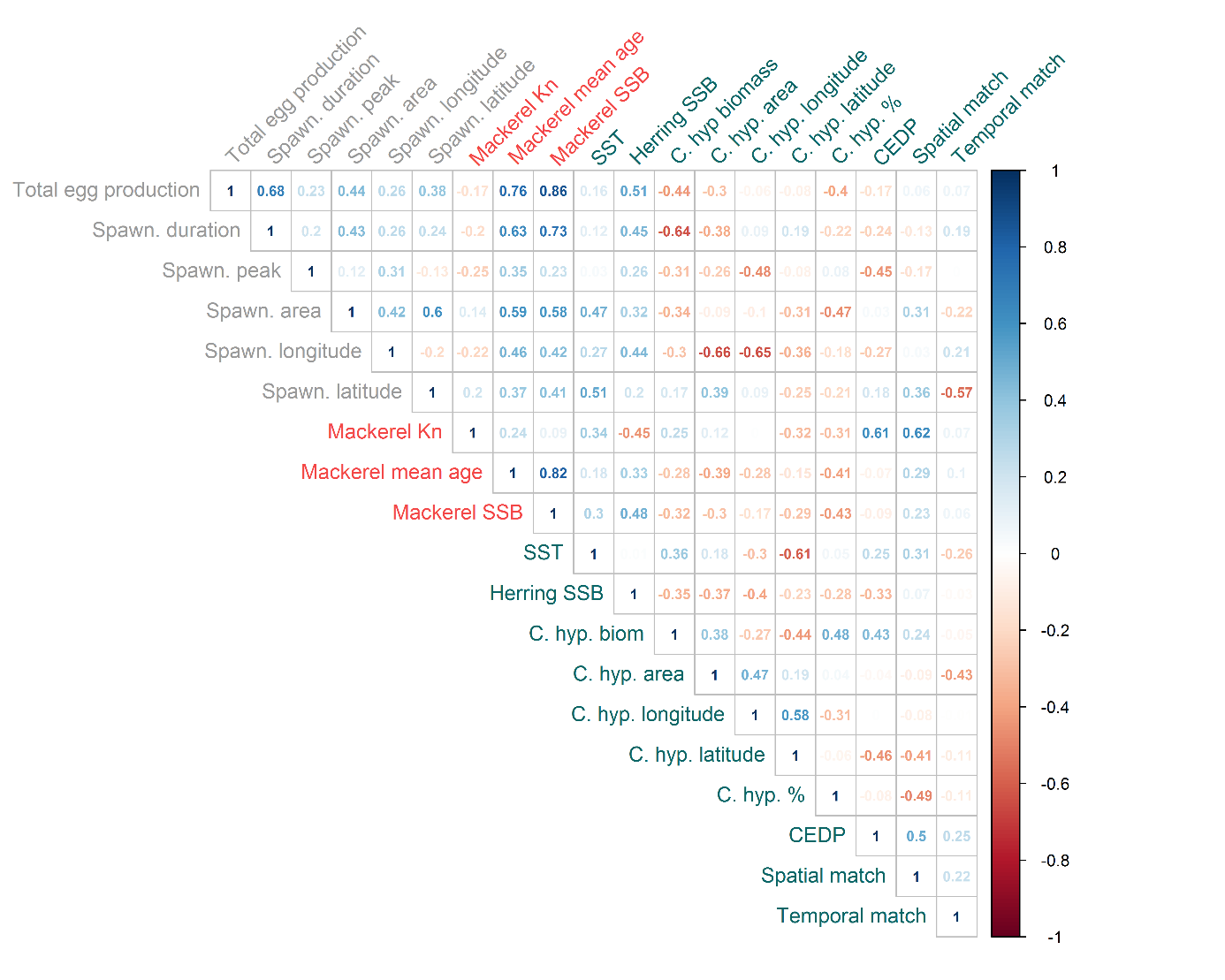


Figure S1. Correlogram representing Pearson’s product-moment correlation (*r*) among all the explanatory variables considered. Red and blue values denote the strength of the negative or positive correlation, respectively. Grey variables: spawning characteristics, red variables: mackerel stock descriptors, green variables: physical and biological environment. Spawning: Sp.; *Calanus hyperboreus*: *C. hyp.*; the percentage of *C. hyperboreus* biomass relative to the total *Calanus* spp. biomass: % *C. hyp.*; mean prey daily egg production: CEDP*.*


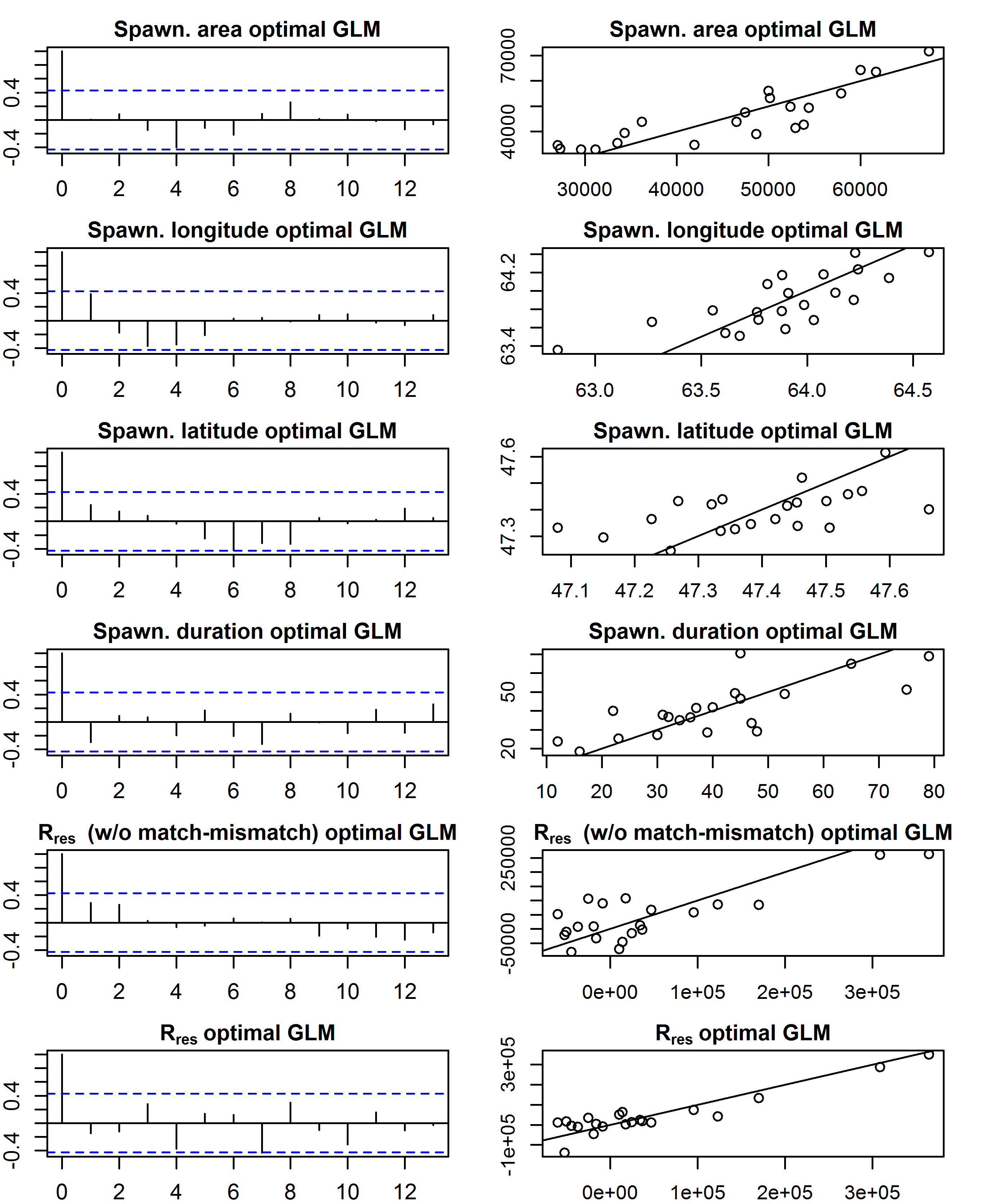


Fig S2. Residuals’ correlograms (left column) and predicted vs observed plots (right column) for all the optimal GLMs obtained in the different steps of the framework.


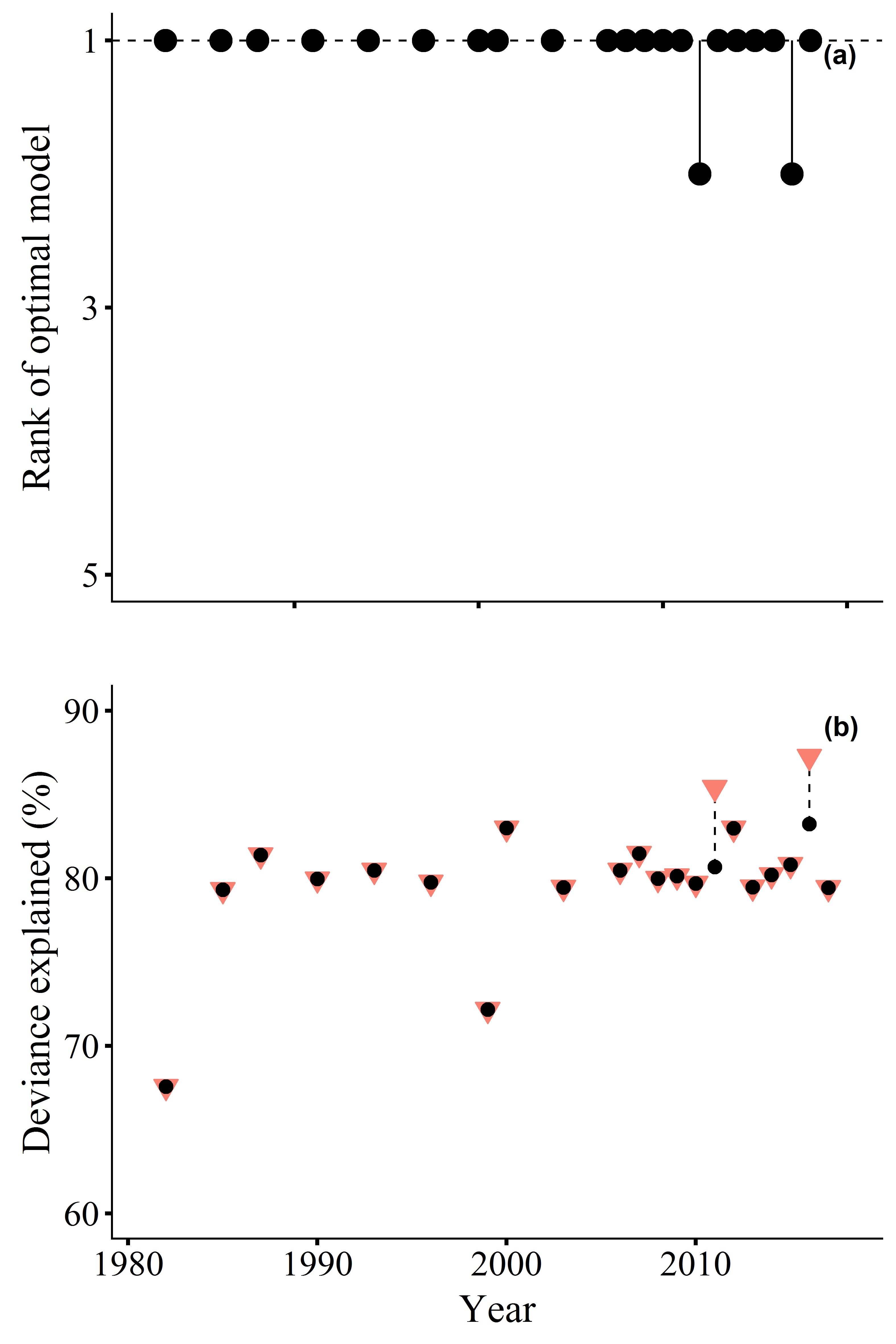


Figure S3: Jackknife analysis of the optimal model for mackerel age-1 recruitment estimated with Beverton-Holt residuals considering the 21 years years with spatial and temporal match data. When a year is omitted from analysis, a) is the corresponding ranking of the optimal model and b) is the corresponding deviance explained by the optimal model (black circle) and the top ranking model (pink triangle).


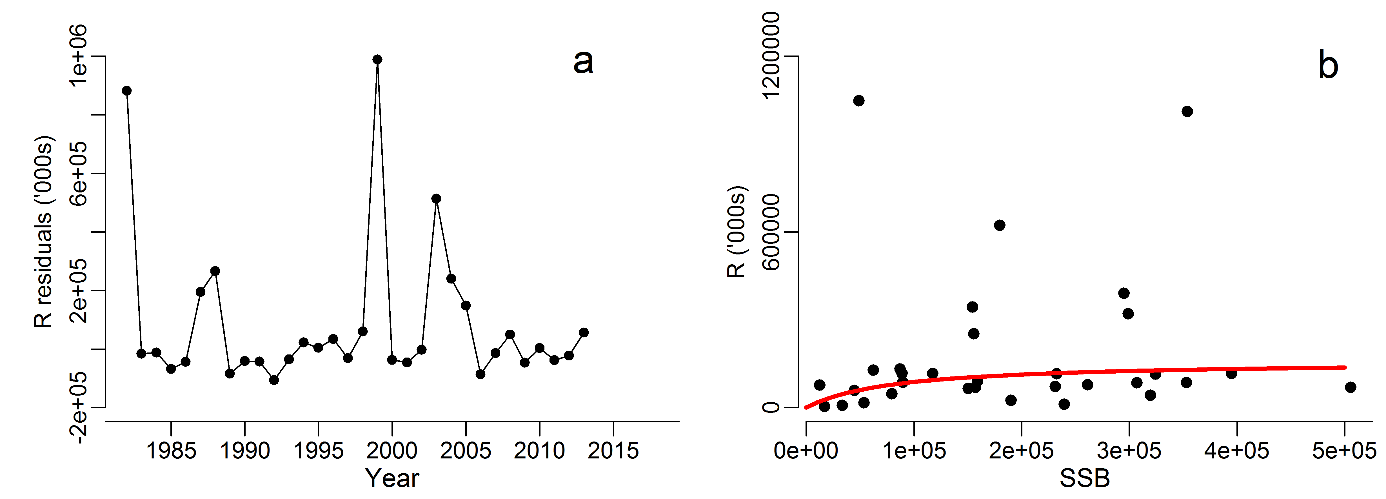


Figure S4. Annual mackerel recruitment residuals (panel a) and the stock-recruitment relationship used to derive recruitment residuals (b) between 1982 and 2013 from the VPA model.

**References used in the SI:**

1. Doniol-Valcroze, T., Van Beveren, E., Légaré, B., Girard, L. & Castonguay, M. *Atlantic mackerel (Scomber scombrus, L.) in NAFO Subareas 3 and 4 in 2016. DFO Can. Sci. Advis. Sec. Res. Doc. 2018/062.* (2019).

2. Van Guelpen, L., Markle, D. F. & Duggan, D. J. An Evaluation of Accuracy, Precision, and Speed of Several Zooplankton Subsampling Techniques. *ICES J. Mar. Sci.* (1982) doi:10.1093/icesjms/40.3.226.

3. Fritzsche, R. A. *Development of fishes of the mid-Atlantic Bight, an atlas of egg, larval and juvenile stages. Vol. V. Chaetodontidae through Ophidiidae. U.S. Fish. Wildl. Serv. Biol. Serv. Program.* (1978).

4. Fahay, M. P. *Early stages of fishes in the Western North Atlantic Ocean (Davis Strait, Southern Greenland and Flemish Cap to Cape Hatteras). Volume one: Acipenseriformes through Syngnathiformes. Northwest Atlantic Fisheries Organization, Dartmouth, N. S.* (2007).

5. Fahay, M. P. *Early stages of fishes in the Western North Atlantic Ocean (Davis Strait, Southern Greenland and Flemish Cap to Cape Hatteras). Volume two: Scorpaeniformes through Tetraodontiformes. Northwest Atlantic Fisheries Organization, Dartmouth, N. S.* (2007).

6. Girard, L. *Identification of mackerel (Scomber scombrus L.) eggs sampled during abundance surveys in the southern Gulf of St. Lawrence. In: The Atlantic mackerel (Scomber scombrus L.) of NAFO Subareas 2 to 6. DFO Can. Sci. Advis. Sec. Res. Doc. 2000/021.* (2000).

7. Therriault, J.-C. *et al.* . *Proposal for a northwest Atlantic zonal monitoring program. Can. Tech. Rep. Hydrogr. Ocean S*. (1998).

8. Runge, J. A. & de Lafontaine, Y. Characterization of the pelagic ecosystem in surface waters of the northern Gulf of St. Lawrence in early summer: The larval redfish-Calanus-microplankton interaction. *Fish. Oceanogr.* **5**, 21–37 (1996).

9. Runge, J. A. & Plourde, S. Fecundity characteristics of *Calanus finmarchicus* in coastal waters of eastern canada. *Ophelia* **44**, 171–187 (1996).

10. McLaren, I. A. & Corkett, C. J. Unusual genetic variation in body size, development times, oil storage, and survivorship in the marine copepod Pseudocalanus. *Biol. Bull.* **155**, 347–359 (1978).

11. Frost, B. The inadequacy of body size as an indicator of niche in the zooplankton. in *Evolution and ecology of zooplankton communities* 743–753 (University of Press of New England, 1980).

12. Peterson, W. T. & Kimmerer, W. J. Processes controlling recruitment of the marine Calanoid copepod *Temora longicornis* in Long Island Sound: Egg production, egg mortality, and cohort survival rates. *Limnol. Oceanogr.* **39**, 1594–1605 (1994).

13. Grégoire, F. & Beaudin, L. *Analytical assessment of the Atlantic mackerel (Scomber scombrus L.) in NAFO Subareas 3-4 in 2013. Secr. can. de consult. sci. du MPO. Doc. de rech. 2014/079*. (2014).
